# Inherited disorder phenotypes: controlled annotation and statistical analysis for knowledge mining from gene lists

**DOI:** 10.1186/1471-2105-6-S4-S18

**Published:** 2005-12-01

**Authors:** Marco Masseroli, Osvaldo Galati, Mauro Manzotti, Karina Gibert, Francesco Pinciroli

**Affiliations:** 1 BioMedical Informatics Laboratory, Bioengineering Department, Politecnico di Milano, piazza Leonardo da Vinci 32, 20133 Milano, Italy; 2 Departament d'Estadística i Investigació Operativa, Universitat Politècnica de Catalunya, C. Pau Gargallo 5, 08028 Barcelona, Spain

## Abstract

**Background:**

Analysis of inherited diseases and their associated phenotypes is of great importance to gain knowledge of underlying genetic interactions and could ultimately give clinically useful insights into disease processes, including complex diseases influenced by multiple genetic loci. Nevertheless, to date few computational contributions have been proposed for this purpose, mainly due to lack of controlled clinical information easily accessible and structured for computational genome-wise analyses. To allow performing phenotype analyses of inherited disorder related genes we implemented new original modules within *GFINDer *, a Web system we previously developed that dynamically aggregates functional annotations of user uploaded gene lists and allows performing their statistical analysis and mining.

**Results:**

New *GFINDer *modules allow annotating large numbers of user classified biomolecular sequence identifiers with morbidity and clinical information, classifying them according to genetic disease phenotypes and their locations of occurrence, and statistically analyzing the obtained classifications. To achieve this we exploited, normalized and structured the information present in textual form in the Clinical Synopsis sections of the Online Mendelian Inheritance in Man (OMIM) databank. Such valuable information delineates numerous signs and symptoms accompanying many genetic diseases and it is divided into phenotype location categories, either by organ system or type of finding.

**Conclusion:**

Supporting phenotype analyses of inherited diseases and biomolecular functional evaluations, *GFINDer *facilitates a genomic approach to the understanding of fundamental biological processes and complex cellular mechanisms underlying patho-physiological phenotypes.

## Background

Understanding clinical phenotypes through their corresponding genotypes is paramount to unveil inherited alterations that can lead to pathological processes and syndromes. However, such comprehension can be very difficult with complex disorders, which frequently present different clinical phenotypes that may result from interactions among multiple and potentially unknown genetic loci. Moreover, considerably different genetic alterations may cause very similar or even the same phenotype [1,2]. Thus, complex and multivariate analyses of the molecular processes underlying phenotypically similar disorders are required to possibly obtain insights into the composite gene and protein interactions [1]. To computationally perform such analyses, numerous structured information and also a few controlled vocabularies that describe biological processes and molecular functions are available [3-6].

Nevertheless, useful clinical information related to genetic diseases is generally not easily accessible and is mainly included in free text descriptions. Hence, it is not suitably organized to be used in computational analyses. This limited availability of controlled structured phenotypic information is hampering the development of effective analytical contributions in the field.

Recently, some tools have been developed to extract genetic and disease information from free text [7-11]. These, which are based on term co-occurrence and association rules or Natural Language Processing techniques, automatically extract sets of genetic and phenotypic related terms. However, due to complexity and variety of clinical biomolecular and genomic descriptions they inherently present extraction errors, with different degrees of precision and recall. Therefore, extracted information should be revised before applying it in subsequent analyses.

In some medical areas, such as oncology, curated phenotypic information of complex genetic disorders is being collected in structured format [12-14]. Nevertheless, currently such data are only available for few classes of diseases and in quantity not yet enough for computational genome-wise analyses.

**Figure 1:**
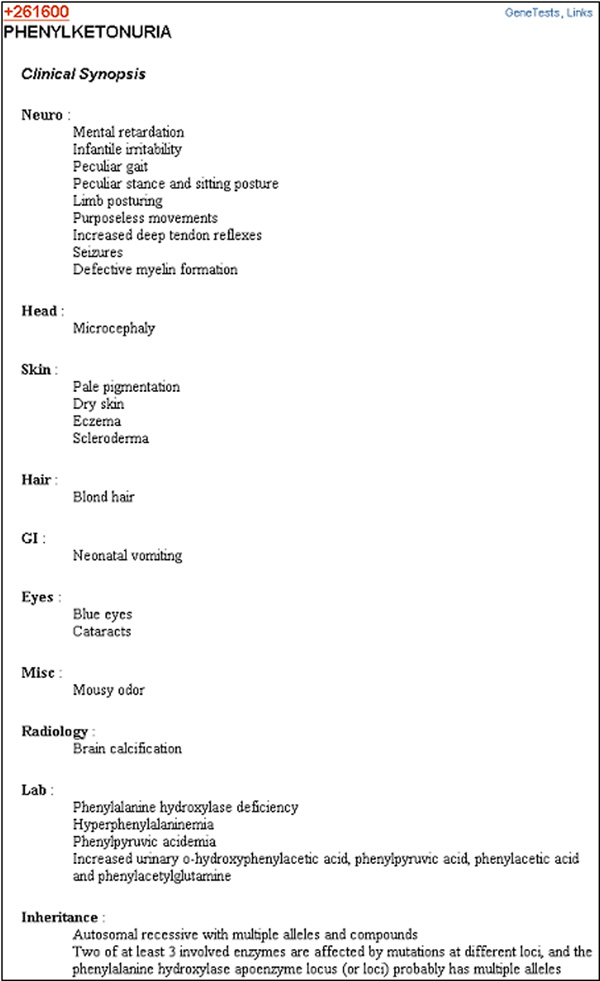
**OMIM Clinical Synopsis section for the Phenylketonuria disease associated with the Phenylalanine Hydroxylase(PAH) human gene and with Mental retardation Neurologic phenotype**. 261600: MIM (Mendelian Inheritance in Man) ID of the Phenylketonuria disease; +: in OMIM a plus sign before a MIM number entry indicates that the entry contains the description of a gene of known sequence and a phenotype; Neuro: Neurologic, GI: Gastrointestinal, Misc: Miscellaneous, Lab: Laboratory phenotype locations.

Thus, at present the most important curated, comprehensive, reliable, and updated source of information in human genetics still remains the Online Mendelian Inheritance in Man (OMIM) databank [15,16], which consist of about 16,100 detailed entries on human genes and genetic disorders. Although it mainly contains free text descriptions on genetic loci, inheritance patterns, allelic variants, biochemical and clinical features, and molecular and population genetics, many OMIM entries also include a Clinical Synopsis section that, in structured text format, outlines disease accompanying signs and symptoms (i.e. phenotypes) and their locations (Figure 1). Regrettably, due to the diversity of presentation of human diseases, and also possibly because OMIM has been in development for decades, information in the Clinical Synopsis sections is not represented in a uniform manner. No controlled vocabulary is used for phenotype and location names. Several typing errors and synonyms for the same name, and different names for overlapping concepts are often present for phenotype location categories (e.g. "Vacular" and "Vascular", "Pre-DX" and "Prenatal diagnosis", or "Growth" and "Development"), as well as for specific phenotypes (e.g. "Vestibular function defect" and "Vestibular dysfunction", "Duplication of great toes" and "Duplicated halluces", or "Hypoplastic digits" and "Digital hypoplasia"), which additionally include a high number of complex and specific findings. Such variability of used descriptive names precludes their direct use in automatic genomic analyses.

To effectively exploit the valuable information in the OMIM Clinical Synopsis section, we first extracted phenotype and their location names and normalized them to create a term vocabulary describing phenotype and phenotype location categories. Then, we hierarchically structured these category descriptions according to increasing detail or topological levels. Finally, within *GFINDer*, a Web system we previously developed for analyzing dynamically aggregated annotations of user uploaded gene lists [17], we used the normalized and structured Clinical Synopsis vocabularies as basis for new *GFINDer *modules specifically devoted to the analysis of inherited disorder related genes. These new modules allow annotating large numbers of user classified biomolecular sequence identifiers with morbidity and clinical information, classifying them according to genetic disease phenotypes and their locations of occurrence, and statistically analyzing the obtained classifications.

## Results

### Normalization and structuring of genetic disease phenotype and location terms

At time of writing, OMIM contained 16,199 entries, 4,570 of them included a Clinical Synopsis section orderly divided into phenotype location categories representing an organ system (e.g. "Endocrine", "Gastrointestinal", "Respiratory", "Skeletal") or a type of finding (e.g. "Laboratory", "Prenatal manifestation"). Under a location category of a specific OMIM genetic disease or locus entry, they are listed the phenotype manifestations in that location known to be associated with the entry. In the OMIM Clinical Synopsis sections we found 169 different names of phenotype location categories and 22,609 distinct phenotype descriptions. After location name normalization, we had 94 unique location category terms and 44 synonyms or typographical errors for 32 category terms. This was obtained by combining, for example, "Lab", "Laboratory abnormalities" and "Laboratory" categories into a unique "Laboratory" category, "Neuro" and "Neurologic" into the single "Neurologic" category, and by merging each of the "Ears", "Eyes", "Joints", "Lungs", "Muscles", and "Limbs" category names with its correspondent singular category term, respectively. Phenotype description normalization produced 20,132 unique controlled descriptions, which included 7,898 descriptions internally subdivided into up to six hierarchical levels of increasing detail. For example, the phenotype descriptions "Hearing loss, sensorineural, non-progressive, non-syndromic, congenital, greater mid frequency than low- or high-frequency loss", "Dysplasia, metaphyseal, marked, of tubular bones, in childhood", "Deafness, sensorineural, prelingual, profound", "Temperature insensitivity, distal, in some patients", and "Hemolytic anemia, following ingestion of fava beans" were subdivided and hierarchically structured into six, five, four, three, and two hierarchical levels, respectively (Table 1).

Similarly, the normalized 94 unique phenotype locations resulted hierarchically structured in three topological levels according to their anatomical organization. The main of such hierarchical levels, which includes broader organ systems or sites, comprised 36 locations.

All the above normalizations and hierarchical structuring produced a total of 33,338 phenotype location entries and 49,072 specific phenotype entries for the available 4,570 OMIM entries with a Clinical Synopsis, which were annotated to 11,433 distinct genes.

### Analysis of inherited disorder phenotypes

**Figure 2:**
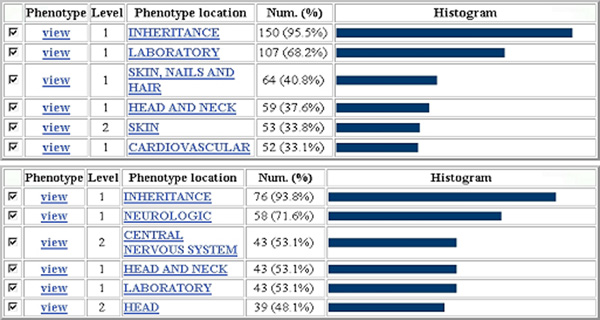
***GFINDer Exploration Genetic Disorders *module: phenotype location categories related to the considered cardiovascular system and neurobiology genes, respectively**. Phenotype view: link to the list of considered genes associated with phenotypes in the specific Phenotype location; Level: level in the defined phenotype location hierarchy (higher levels correspond to more specific locations); Num. (%): absolute and percentage number of considered genes associated with phenotypes in the specific phenotype location.

**Figure 3:**
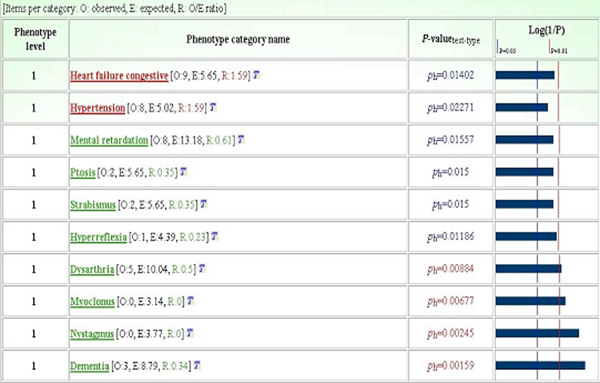
***GFINDer Statistics Genetic Disorders *module: phenotypes most significantly over- and under-represented in the considered cardiovascular system versus neurobiology gene classes**. Phenotype level: level in the defined phenotype hierarchy (higher levels correspond to more detailed and specific phenotype descriptions); *P*-value_test-type_: *P *value defining association between a given phenotype and a considered class of genes, and initial of used statistical test name (h: hypergeometric distribution test).

*GFINDer *[17] is a Web system we previously implemented that dynamically aggregates updated functional annotations of user-uploaded gene lists from different databanks and allows performing their comprehensive explorations and statistical analyses. To this aim, *GFINDer *is organized in independent and interconnected modules that use several controlled vocabularies describing gene related biomolecular processes and functions. Within *GFINDer*, we developed new *Genetic Disorder Annotation*, *Exploration*, and *Statistics *modules that exploit the phenotype and phenotype location vocabularies OMIM provides and which we normalized and structured. The *Annotation *module produces a tabular output of user-uploaded gene IDs enriched with related genetic disease phenotypes and phenotype locations, and with several other annotations automatically retrieved from many different databanks. The *Exploration Genetic Disorders *module (Figure 2) allows to easily and graphically understand either how many and which phenotype locations and their specific signs and symptoms are correlated to each considered gene, or how many of the selected genes refer to each of those locations, or phenotypes. When uploaded genes are subdivided in classes (e.g. from clustering analysis of microarray assay results), the *Statistics Genetic Disorders *module allows to estimate relevance of OMIM controlled annotations for the uploaded classes of genes by highlighting phenotypic locations and their specific signs and symptoms significantly more represented within the user-defined classes of genes. To this aim, the annotated genes are grouped accordingly to their class and annotation categories, and their distribution among the considered categories is statistically evaluated. For this purpose, different statistical tests and type of corrections for multiple tests have been implemented in *GFINDer*, as following described in the Methods section. After selecting a specific gene class, for each inherited disorder annotation category in that class the module provides the observed number of input genes, their expected number, and the significance *p*-value for that category with its histogram (Figure 3). External links to the OMIM descriptions and Clinical Synopsis sections related to the considered genes are also given.

### Validation of implemented application

To assess capabilities of implemented *GFINDer Genetic Disorders *modules, we used them to evaluate a set of 1,046 human clones spotted on the 7734-1 or 7736-1 Clontech microarrays [18] focused on cardiovascular system (522 clones) and neurobiology (524 clones), respectively. Using *GFINDer Annotation *module we found these clones corresponded to 935 distinct genes. Out of them, 271 (250 autosomal and 21 X-linked) were involved in 462 inherited diseases, and 122 (97 autosomal and 25 X-linked) were associated with 679 different phenotypes in 63 locations. Therefore, we used the *GFINDer Exploration *and *Statistics Genetic Disorders *module to evaluate the relevant presence of genes associated with specific inherited disease phenotypes or locations within the considered cardiovascular system correlated genes (CARDIO) versus the neurobiology related genes (NEURO).

With the *Exploration *module we observed the distribution of phenotypes and phenotype locations (Figure 2) within the two considered CARDIO and NEURO classes of genes. Then, using the *Statistics *module we evaluated the phenotype locations most represented in the CARDIO versus NEURO class. We concentrated only on genes with phenotype location annotations and on location categories associated with at least two of the considered genes. Statistical analysis correctly selected phenotype locations related to the appropriate class of considered genes. In fact, the significant selected locations included "Cardiovascular" (*p *= 0.00074), "Heme" (*p *= 0.02206), "Heart" (*p *= 0.03165) and "Cardiac" (*p *= 0.03562) categories for the CARDIO class, and "Neurologic" (*p *< 0.00001), "Central nervous system" (*p *= 0.0002), "Behavioural/Psychiatric manifestations" (*p *= 0.00347), "Peripheral nervous system" (*p *= 0.01702) categories for the NEURO class.

Finally, we analyzed the phenotypes most represented in the CARDIO versus NEURO class. Focusing only on genes with phenotype annotations and on phenotype categories associated with at least two of the considered genes, *GFINDer *statistical analysis properly highlighted as most relevant in each gene class signs and symptoms logically pertaining to that class (Figure 3). In fact, apart from inheritance pattern phenotypes, the CARDIO gene class included "Heart failure congestive" (*p *= 0.01402) and "Hypertension" (*p *= 0.02271) phenotypes, whereas the NEURO class included "Dementia" (*p *= 0.00159), "Myoclonus" (*p *= 0.00677), "Dysarthria" (*p *= 0.00884) and "Mental retardation" (*p *= 0.01557) phenotypes.

Obtained results demonstrate validity of the approach for the analysis of genetic disorder phenotypes, locations and related genes that we developed, implemented and made available within the *GFINDer *Web system.

## Discussion

Our efforts to derive from the OMIM entries a controlled vocabulary of phenotype locations and descriptions enabled us to normalize and structure the valuable OMIM phenotypic data according to the obtained vocabulary and make them suitable for computational use. Although detailed phenotype descriptions could be further homogenized and standardized, their subdivision in hierarchical levels of detail that we performed allows to group specific phenotypes according to their common general traits, without loosing their specific characteristics. So, for example "Mental retardation, moderate" and "Mental retardation, nonspecific" can be both generally considered as "Mental retardation" and at the same time they can be treated as different types of mental defects. This provides the chance to modulate analysis granularity when searching for phenotypic traits shared among multiple diseases or genotypes. It also ensures more significant and clear results when categorical statistical analyses are performed at lower granularity levels of detail. Such interesting feature, proper of the hierarchical structure and hence belonging also to the defined phenotype location hierarchy, is exploited in the new *GFINDer Genetic Disorders *modules implemented for the study of genetic disorder related genes.

In the *Exploration *module, the user can select the detail level of phenotype description, or topological location, at which exploring the genetic disorder phenotypic annotations associated with a considered set of genes, or explore all levels at the same time (Figure 2). In the *Statistics *module, consecutive statistical tests are executed on each categorical annotation independently on its level of detail. Then, analysis results are shown listing each tested categorical annotation with its hierarchical level and the obtained *p*-value (Figure 3). This simultaneously provides a comprehensive view of the statistical significance of all considered annotations and clearly highlights the phenotypic characteristics with lowest *p*-value within each of the considered user-defined classes of genes, specifying also their granularity level. Validation results showed that the above is correctly performed also when genes in different considered classes are associated with the same genetic disorder phenotypes, as it happens for disorders that may have a cardiovascular and neurological etiology. In these cases, although obtained *p*-values do not reach statistical significance, lower *p*-values properly indicate more relevant phenotypic characteristics (Figure 3).

*GFINDer*, which is freely available on-line for non-profit use at , is hence a unique valuable tool that provides support for a phenotypic taxonomy of inherited diseases. Although several tools are available for the analysis of gene annotations according to the Gene Ontology and few other controlled vocabularies, at present to our knowledge none supports phenotype analyses.

## Conclusion

Our normalization and structuring of the valuable phenotypic information that OMIM offers generated a controlled phenotypic vocabulary suitable for computational purposes. As our validation demonstrated, its use within the new-implemented *GFINDer *modules allows effective phenotypic analyses of inherited disorders related genes. The new *GFINDer *functionalities can hence help in better interpreting high-throughput gene lists and in unveiling new biomedical knowledge about the considered genes. Thus, they can facilitate a genomic approach in the understanding of fundamental biological processes and complex cellular mechanisms, underlying patho-physiological phenotypes, through their corresponding genotypes.

## Methods

### Normalization and structuring of genetic disease phenotype and location terms

From the *omim.txt *file, which contains the entire free text of the OMIM databank and is freely available from the OMIM FTP site [19], by using standard text-parsing procedures we extracted phenotype descriptions and phenotype location names included in its Clinical Synopsis sections. Then, the isolated names and descriptions were visually inspected and normalized. A unique term was assigned to each location synonym or incorrect written name. Phenotype descriptions were corrected for typo errors and different descriptions for the same sign or symptom were uniformed to the same most recurrent or most correct description. Furthermore, when phenotype descriptions included general illustrative terms together with more specific traits, we subdivided them into hierarchical levels according to their increasing degree of detail. Similarly, we hierarchically structured also phenotype locations. In the available OMIM Clinical Synopsis sections, phenotype locations are partially and inconsistently structured according to their topological organization (Figure 1). After location term normalization, we homogenized the provided structure and organized it in hierarchical levels in relation to the anatomical organization of the described location categories.

### Implemented *GFINDer *architecture for phenotype annotation analyses

*GFINDer *Web system is implemented in a three-tier architecture based on a multi-database structure. In the first tier, the *data tier*, a MySQL DBMS manages all considered genomic annotations stored in different relational databases. In this tier, we added a specifically designed relational database where we hierarchically structured information about genetic disease phenotypes by exploiting the multi-granular level and topological descriptions of OMIM Clinical Synopsis phenotypes and phenotype locations, respectively. To associate an inherited disorder phenotype with the involved genes or genetic loci, if any, we considered the MIM codes associated with a gene, as provided by the Entrez Gene database [3]. Using Java programming language, we implemented procedures able to automatically import and keep updated, in the *GFINDer data tier*, genetic disorder phenotype information and correspondent gene annotations, as soon as new releases of them become available in OMIM and Entrez Gene databanks. According to the defined controlled vocabulary of phenotype descriptions and phenotype location terms, specific procedures automatically normalize and structure previously revised annotations included in the latest imported release of OMIM Clinical Synopsis information. When novel Clinical Synopsis annotations are included in a new imported release, an advisory email is automatically sent to the *GFINDer *phenotype vocabulary supervisor who can use specifically designed *GFINDer *Web interfaces to review and, if required, normalize and structure the retrieved new annotations.

In *GFINDer processing tier *we used Microsoft ActiveX Data Object technology and Standard Query Language to interact with the MySQL DBMS server on the *data tier*, and implemented management and analysis routines in Javascript and Active Server Page scripts. Created analysis procedures include statistical categorical analyses based on the normalized lists of unique phenotype and location category terms. They employ different tests to assess statistical significance of the over and under representation of categorical phenotypic annotations in a group of user-classified genes.

In *GFINDer user tier*, which is composed of any client computer connected to the Web server on the *processing tier *through an Internet/intranet communication network, we employed Hyper Text Markup Language and Javascript to implement a Web graphic interface for the new developed *Genetic Disorder *modules.

### Statistical analysis

Statistical techniques were used in our approach implemented in *GFINDer *for analyzing genetic disorder phenotypes and their locations. Because a gene may or may not be associated with a certain phenotype category defined in the used controlled vocabulary, the number of genes and their frequency, distribution, and probability of occurrence is calculated for each phenotype category related to a considered gene set. Several different statistical tests can be used to calculate a probability value of having x genes or fewer associated with a given phenotype category. In *GFINDer *the *hypergeometric test *(more time-consuming), the *binomial test *(which is an asymptotic limit of the first for high number of genes), and the *exact Fisher test *(based on a two-way table crossing gene classes and phenotype categories) were implemented [17,20,21]. As usual in all significance tests, small *p*-values relate to relevant phenotype categories for a certain class of genes. However, depending on the number of considered genes and their associated phenotype annotations, the number of performed statistical tests can be high. This can greatly increase the Type-I error associated to the tests, i.e. the probability of obtaining a significant *p*-value by chance when the null hypothesis is true (or the false-positive value, as it is known in the medical field). This requires corrections on the calculated *p*-values in order to obtain proper significances.

In *GFINDer *several correction methods for multiple tests have been included. The simplest and most strict is the Bonferroni method that can be applied if the performed tests are independent [22]. It consists on changing the threshold α of each single test, from which every corresponding *p*-value of each test is considered significant, in such a way that the Type-I error of the whole set of tests is maintained. The correction is the following α_corrected _= α / N, where N is the number of performed tests. From a practical point of view this is equivalent to keep the usual threshold α for the performed tests and apply a correction to the observed *p*-values such that *p*_corrected _= N * *p*. However, the Bonferroni method greatly reduces the power of detecting a specific hypothesis when the number of tests increases. False Discovery Rate (FDR) and Family-Wise Error-rate (FWE), an extension of Bonferroni method, are milder corrections and they are even suitable when independence among tests does not hold [23]. The former briefly consists in ordering the N *p*-values such that the maximum has rank N and the minimum has rank 1. Then, the correction to be applied is *p*_corrected _= p * N / rank(*p*), except for the maximum *p*-value that is not corrected. The latter instead, in the implementation proposed by Benjamini and Hochberg [23], uses the following *p*-value correction: *p*_corrected _= p * (N - rank(*p*) + 1). All three methods above illustrated are available in *GFINDer*. Among them the FDR, which is the mildest of the three and practically consists in defining the maximum acceptable number of obtained false-positive tests, is considered the most suitable correction method to be applied on genomic data.

## Authors' contributions

MMas was responsible for the overall project conception and coordination, was involved in design and testing of *GFINDer *software modules, and wrote this manuscript. OG developed *GFINDer *software modules devoted to the analysis of inherited disorders, and was involved in their design and testing. MMan visually inspected and normalized the OMIM phenotype vocabulary. KG supervised the statistical analysis aspects of the project. FP provided supervision and funding of the project.

## Figures and Tables

**Table 1:** Hierarchical structure of some of the phenotype categories considered in *GFINDer*, as derived from the correspondent phenotype descriptions provided by OMIM databank.

**Phenotype**
Aphasia
Biliary atresia
extrahepatic
Bleeding diathesis
Cardiomyopathy
Coarse facies
Deafness
sensorineural
prelingual
profound
Edema
Elevated IgA
Femoral bowing
present at birth
straightening with time
Gastric ulcer
Hallux valgus
Hemolytic anemia
following ingestion of fava beans
Hypertension
Mental retardation
Pain insensitivity
distal
Psychosis
Ptosis
Quadriplegia
episodic
Recurrent sinusitis
Renal failure
reversible
Seizure
Severe ataxia
Temperature insensitivity
distal
in some patients
